# The *Staphylococcus aureus* toxin–antitoxin system YefM–YoeB is associated with antibiotic tolerance and extracellular dependent biofilm formation

**DOI:** 10.5194/jbji-6-241-2021

**Published:** 2021-07-02

**Authors:** Xinyu Qi, Kimberly M. Brothers, Dongzhu Ma, Jonathan B. Mandell, Niles P. Donegan, Ambrose L. Cheung, Anthony R. Richardson, Kenneth L. Urish

**Affiliations:** 1 Arthritis and Arthroplasty Design Group (AAD Lab), Department of Orthopaedic Surgery, College of Medicine, University of Pittsburgh, Pittsburgh, Pennsylvania, USA; 2 Department of Orthopedic Surgery, the First Affiliated Hospital of Traditional Chinese Medicine of Guangzhou University, Guangzhou, Guangdong, China; 3 Department of Microbiology and Immunology, Geisel School of Medicine at Dartmouth, Hanover, NH, New Hampshire, USA; 4 Department of Microbiology and Molecular Genetics, University of Pittsburgh, Pittsburgh, Pennsylvania, USA

## Abstract

The high antibiotic tolerance of *Staphylococcus aureus* biofilms is associated with challenges for treating periprosthetic joint infection. The toxin–antitoxin system, YefM–YoeB, is thought to be a regulator for antibiotic tolerance, but its physiological role is unknown. The objective of this study was to determine the biofilm and antibiotic susceptibility phenotypes associated with *S. aureus* yoeB homologs. We hypothesized the toxin–antitoxin yoeB homologs contribute to biofilm formation and antibiotic susceptibility. Disruption of yoeB1 and yoeB2 resulted in decreased biofilm formation in comparison to Newman and JE2 wild-type (WT) *S. aureus* strains. In comparison to yoeB mutants, both Newman and JE2 WT strains had higher polysaccharide intercellular adhesin (PIA) production. Treatment with sodium metaperiodate increased biofilm formation in Newman WT, indicating biofilm formation may be increased under conditions of oxidative stress. DNase I treatment decreased biofilm formation in Newman WT but not in the absence of yoeB1 or yoeB2. Additionally, WT strains had a higher extracellular DNA (eDNA) content in comparison to yoeB mutants but no differences in biofilm protein content. Moreover, loss of yoeB1 and yoeB2 decreased biofilm survival in both Newman and JE2 strains. Finally, in a neutropenic mouse abscess model, deletion of yoeB1 and yoeB2 resulted in reduced bacterial burden. In conclusion, our data suggest that yoeB1 and yoeB2 are associated with *S. aureus* planktonic growth, extracellular dependent biofilm formation, antibiotic tolerance, and virulence.

## 1 Introduction

Periprosthetic joint infection (PJI) is one of the most challenging complications following total joint arthroplasty. The current treatment strategy involves irrigation and debridement of the infected area as well as administration of intravenous antibiotics. Treatment failure of PJI is high, at around 50 %–60 % (Biau et al., 2010). The major pathogen associated with PJI in the United States is the gram-positive bacteria *Staphylococcus aureus* (Del Pozo, 2018), with up to 50 % of cases involving the extremely difficult to treat methicillin-resistant *S. aureus* (MRSA) (Nodzo et al., 2017).

PJI infections are primarily caused by antibiotic-tolerant biofilms on the surface of the implant (Tande and Patel, 2014; Mooney et al., 2018; Urish et al., 2016; Del Pozo, 2018). These infections are difficult to eradicate and often involve removal of the infected device, creating a huge economic burden on healthcare and impact on the wellbeing and daily life of patients. The scientific literature suggests antibiotic tolerance arises from antibiotic-resistant persister cells, a decreased bacterial metabolism when in the biofilm state, and large production of exopolysaccharide (EPS) that coats the biofilm and prevents drug diffusion to bacteria (Neut et al., 2007).

The mechanisms of *S. aureus* biofilm formation are poorly understood. Bacterial toxin–antitoxin (TA) systems are believed to play a critical role in biofilm antibiotic tolerance and resistance (Wen et al., 2014; Kedzierska and Hayes, 2016; Thomopoulos et al., 2015). This system consists of a toxin that will disrupt a cellular process (translation, etc.) and an antitoxin that prevents toxin activation. Under conditions of environmental stress such as high temperature shock, oxidative stress, and exposure to antibiotics, the antitoxin disassembles, and the toxin becomes activated. This activation leads to disruption in bacterial metabolism, inducing a state of dormancy (Biau et al., 2010; Wang et al., 2012; Fasani and Savageau, 2013). When environmental stressors are no longer present, the antitoxin system is reactivated, binding the toxin, and bacteria are once more susceptible to these stressors.

Probably the most well-studied TA system in *S. aureus* is the type II TA system MazEF. Our previous work identified the role of MazEF in biofilm antibiotic tolerance (Ma et al., 2019). The second type II TA system in *S. aureus* is YefM–YoeB, a ribosome-dependent RNase that cleaves close to the start codon (Schuster and Bertram, 2016). YoeB is the toxin, and YefM is the antitoxin. In contrast to the MazEF system that only has one toxin MazF, there are two types of yoeB toxin genes: yoeB1 and yoeB2 (Chan et al., 2012). The YefM antitoxin inhibits the toxin YoeB through protein–protein interactions (Schuster and Bertram, 2016). Overproduction of YoeB inhibits *Streptococcus pneumoniae* cell growth and viability (Bakar et al., 2015); however, it is still unclear how yoeB1 and yoeB2 affect *S. aureus* growth and biofilm formation.

The objective of this study was to identify the phenotype and physiological role of *S. aureus* yoeB in planktonic growth, biofilm formation, antibiotic susceptibility, and virulence.

## 2 Materials and methods

For this study to measure differences between the wild type (WT) and yoeB mutants, we conducted 12 different experiments in two different strain backgrounds: (1) planktonic growth assays, (2) biofilm bacterial burden assays, (3) polysaccharide intracellular adhesion (PIA) quantification, (4) biofilm quantitation after treatment with sodium metaperiodate, (5) biofilm extracellular DNA quantification (eDNA), (6) biofilm quantitation after DNAse I treatment, (7) biofilm extracellular protein quantification, (8) biofilm quantitation after proteinase K treatment, (9) MIC (minimum inhibitory concentration) determination, (10) planktonic antibiotic susceptibility assays, (11) biofilm antibiotic susceptibility assays, and (12) in vivo infection in a murine abscess model. All experiments have been divided into the major headings listed below.

### 2.1 Bacterial strains and growth conditions

Newman WT, yoeB1, and yoeB2 *Staphylococcus aureus* deficient mutants were kindly provided by Niles Donegan. The WT USA300 JE2 strain was purchased from the American Type Culture Collection (ATCC). The USA300 JE2 strain yoeB1 and yoeB2 mutants came from the Nebraska Transposon Mutant Library. All *S. aureus* cultures were grown overnight in trypticase soy broth (TSB) medium at 37 ${}^{\circ }$C with shaking.

### 2.2 Planktonic growth assay

Bacterial growth assays were performed according to Kato et al. (2017). Briefly, after 16 h incubation, an overnight culture of the WT reference strain and yoeB mutants (Newman and USA300 JE2 strain backgrounds) was grown as described in Sect. 2.1 and normalized using a 0.5 McFarland standard (Hardy Diagnostics). Normalized cultures were diluted to $1\times 10^{7}$ colony-forming units (CFU) in TSB medium. An amount of 100 µL of each diluted bacterial suspension was added to a 96-well plate and incubated at 37 ${}^{\circ }$C. Absorbance at OD${}_{600}$ was measured every hour for 6 h with a Multiskan plate reader (Infinite 200 Pro, Tecan). Results are expressed as a mean value for each time point ± standard deviation.

### 2.3 Biofilm bacterial burden assay

Sterile titanium rods (10mm×1mm) were placed into a six-well plate (Costar, USA) containing 4 mL TSB medium. Overnight cultures were grown as described in Sect. 2.1 and normalized to $1\times 10^{5}$ CFU/mL using the 0.5 McFarland standard as described in Sect. 2.2. To form mature biofilms, plates were incubated for 24 to 72 h at 37 ${}^{\circ }$C. Wells were replaced with TSB medium every 24 h to remove any planktonic bacteria. At the experimental endpoints (24, 48, or 72 h), titanium rods were washed three times in PBS, sonicated, and plated onto TSA II blood agar plates (Thermo Fisher Scientific, USA) to enumerate bacteria by CFU. Results are expressed as a mean value ± standard deviation.

### 2.4 Biofilm polysaccharide intracellular adhesin (PIA) quantification and changes in biofilm formation after sodium metaperiodate treatment

PIA detection was performed as described by Cerca et al. (2006). Biofilm samples were grown and collected following the PBS washing step as described in Sect. 2.3. Samples were resuspended in 50 µL of 0.5 M EDTA (pH 8.0) and incubated for 5 min at 100 ${}^{\circ }$C. Next, samples were centrifuged for 10 min at 5000× g. An amount of 40 µL of the supernatant was collected and incubated with 10 µL proteinase K (20 mg/mL) for 30 min at 37 ${}^{\circ }$C. Samples were then mixed with 10 µL of 20 mM Tris pH 7.4, 150 mM NaCl, and 0.1 % bromophenol blue. An amount of 3 µL of the preparation and dilutions was spotted onto a nitrocellulose filter, blocked with 3 % BSA in TBS 0.1 % Tween (TBST) for 2 h at room temperature. Then samples were incubated with PIA antibody (L3892; Sigma) overnight at 4 ${}^{\circ }$C. The filter was washed five times in TBST. PIA was detected by ECL chemiluminescent detection reagent (Thermo Scientific) according to the manufacturer's instructions. Images were developed and quantified using a ChemiDoc Touch imaging system (Bio-Rad). In an independent biofilm assay, bacteria were treated with 10 µM sodium metaperiodate or PBS 2 h prior to experimental endpoints. Crystal violet was used to stain the biofilm, then it was dissolved in 30 % acetic acid. The absorbance of the biofilm was measured at 600 nm. All results are expressed as a mean value ± standard deviation.

### 2.5 Biofilm extracellular DNA quantification (eDNA) and changes in biofilm formation after DNase treatment

WT and yoeB mutant mature biofilms were grown on titanium rods and harvested as described in Sect. 2.3. For eDNA quantification, biofilm samples were treated with 5 µg/mL proteinase K and 20 µg/mL N-glycanase for 1 h at 37 ${}^{\circ }$C. Samples were filter-sterilized with a 0.45 µM filter (Fisher Scientific). The filtered resuspension was diluted 1:2 with 2 µM SYTOX green (Thermo Fisher Scientific). Fluorescence was measured using a Multiskan plate reader (Infinite 200 Pro, Tecan), with excitation and emission wavelengths of 465 and 510 nm, respectively. The amounts of eDNA relative to wild-type Newman or USA300-JE2 were calculated. To analyze any resulting changes in biofilm formation after DNase treatment, biofilms were grown and washed as described in Sect. 2.3. A period of 2 h prior to quantification, biofilms were treated with 100 U/mL DNase I or PBS. Crystal violet staining was performed and quantified as described in Sect. 2. All results are expressed as a mean value ± standard deviation.

### 2.6 Biofilm protein quantification and changes in biofilm formation after proteinase K treatment

Biofilms were grown and harvested as described in Sect. 2.3. Biofilm protein concentration was determined with a bicinchoninic acid (BCA) assay reagent kit (Thermo Scientific) according to the manufacturer's specifications. Prior to protein quantification, biofilms were treated with 5 µg/mL proteinase K or PBS 2 h before the BCA assay. In a separate biofilm assay, crystal violet staining and quantification as described in Sect. 2.4 was used to quantify any changes in biofilm after proteinase K treatment. All results are expressed as a mean value ± standard deviation.

### 2.7 Minimum inhibitory concentration (MIC) assay

MIC assays were performed according to the manufacturer's instructions. Overnight cultures were diluted in TSB medium to achieve a specified inoculum turbidity by normalizing OD${}_{600}$ absorbance to a 0.5 McFarland standard ($∼1.5\times 10^{8}$ colony-forming unit (CFU)/mL bacteria). The diluted bacteria were plated onto a blood agar plate using a plate spreader to evenly distribute the inoculum. Plates were air-dried for 20 min at room temperature. Next, a cefazolin or vancomycin Etest strip (Liofilchem, Italy) was applied to the agar plate. Plates were incubated overnight at 37 ${}^{\circ }$C. After a 24 h incubation, MIC values were read and recorded.

### 2.8 Planktonic susceptibility assay

Cefazolin and vancomycin were purchased from Sigma Aldrich (USA) for antibiotic susceptibility assays. *S. aureus* strains and mutants were cultured in TSB medium overnight, normalized to the same 0.5 McFarland standard as described above, diluted to $1\times 10^{7}$ CFU, and grown for 16 h at 37 ${}^{\circ }$C. Pre-treatment bacterial concentrations were determined by CFU assay on TSA II plates. Next, 10X MIC of cefazolin and vancomycin were added to the inoculum for all strains, incubated at 37 ${}^{\circ }$C with shaking for 24 h, and plated to identify surviving bacteria by CFU assay. Percent planktonic cell survival was calculated and compared to pre-treatment CFU values. Results are expressed as percent survival ± standard deviation.

### 2.9 Biofilm susceptibility assay

For biofilm susceptibility assays, sterile titanium rods for biofilm formation were prepared as described above. In order to grow mature biofilm, bacteria were incubated for 72 h to quantify the bacterial burden prior to antibiotic treatment. The remaining titanium rods were treated with 10X MIC of vancomycin or cefazolin calculated from planktonic cultures and incubated for an additional 48 h. Plates were replaced with media and antibiotics every 24 h. After 48 h, titanium rods were removed, washed in PBS, sonicated, and plated as described above. Biofilm percent survival was calculated and compared to the pre-treatment CFUs. Results are expressed as a percent survival ± standard deviation.

### 2.10 In vivo infection in a murine abscess model

Based on our previous work (Ma et al., 2019), *S. aureus* USA300-JE2 strain background was selected for in vivo experiments. A number of 8- to 12-week-old C57BL/6 mice were purchased from the Jackson laboratory (Bar Harbor, ME). All animal protocols used for these experiments were approved by the University of Pittsburgh's Institutional Animal Care and Use Committee (IACUC). All experiments were performed in accordance with relevant guidelines and regulations. To create a model for neutropenia, mice were injected with 100 µL cyclophosphamide (150 mg/kg 3 d prior to infection and 100 mg/kg 1 d prior to infection). After anesthetizing the mice with 2 % isoflurane, leg hair was removed and washed with 3 % betadine. An amount of 100 µL of $1\times 10^{6}$ CFU of JE2-WT, JE2-yoeB1, or JE2-yoeB2 was injected into the thigh to create an abscess (n=8 per group). All mice were monitored for any signs of lack of grooming, loss of appetite, dehydration, weight loss, swelling, or signs of sepsis until experimental endpoints. At 72 h (3 d) post-inoculation, animals were euthanized. A ∼5×5 mm piece of thigh muscle from the infection area was obtained and placed in 1 % PBS tween (PBST) on ice. Abscess bacterial burden was used as a measure of virulence (Kobayashi et al., 2015). In order to quantify bacterial burden in CFU/mL, infected samples were sonicated for 10 min, serially diluted 1:10 in PBS, plated onto blood agar, and incubated overnight at 37 ${}^{\circ }$C. Results are expressed as a mean value ± standard deviation.

### 2.11 Statistical analysis

Statistical analysis was based on the number of populations and comparisons. A paired Student t test was used for two populations. One-way analysis of variance (ANOVA) and Tukey's post hoc analysis with alpha set to 0.05 were used for multiple comparisons. Repeated measure analysis was used for analysis of differences over time. For all statistical tests used, p values <0.05 were considered statistically significant.

**Figure 1:**
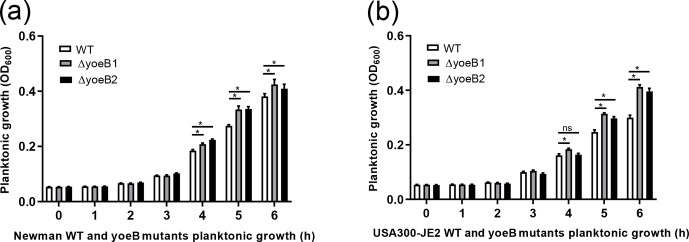
YoeB suppresses *S. aureus* planktonic growth. **(a)** Newman and **(b)** JE2 (ns: not significant, * p<0.05).

## 3 Results

### 3.1 Planktonic growth assay

In both strain backgrounds, increased planktonic growth in yoeB mutants in comparison to WT was observed in the early log phase (at 5 and 6 h) (Fig. 1). In the Newman strain, differences between WT and yoeB mutants were not observed until the 4 h time point, with a mean OD${}_{600}$ of 0.20 and 0.22 for yoeB mutants in comparison to WT at a mean of 0.18 (Fig. 1a, p<0.05). These differences became more evident by the 5 h time point, with a mean of 0.27 for WT, 0.33 for yoeB1, and 0.34 for yoeB2 (Fig. 1a, p<0.05). At 6 h, the differences were even greater, with a mean of 0.38 for WT, 0.43 for yoeB1, and 0.41 for yoeB2 (Fig. 1a, p<0.05). In the JE2 strain, differences in bacterial growth were first observed between WT and yoeB1 by the fourth hour of growth, with a mean of 0.16 for the WT and a mean of 0.18 for yoeB1 (Fig. 1b, p<0.05). Differences between WT and both yoeB mutants occurred starting at the 5 h time point, with a mean of 0.25 for the WT, 0.31 for yoeB1, and 0.31 for yoeB2 (Fig. 1b, p<0.05). At the 6 h time point, the WT had a mean of 0.30, yoeB1 0.41, and yoeB2 0.40 (Fig. 1b, p<0.05).

**Figure 2:**
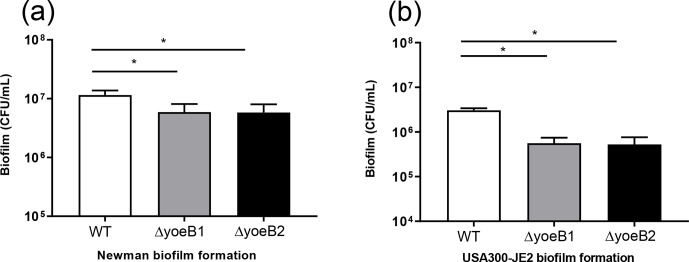
Loss of yoeB decreases biofilm. **(a)** Newman and **(b)** USA300-JE2 bacterial burden (CFU/mL) (* p<0.05).

### 3.2 Biofilm bacterial burden assay

When bacterial burden of biofilm growth after 72 h (day 3) in the Newman strain background was measured, yoeB1 and yoeB2 had a decrease in biofilm formation, with a mean CFU/mL of $5.92\times 10^{6}$ and $5.80\times 10^{6}$ respectively in comparison to WT at $1.2\times 10^{7}$ CFU/mL (Fig. 2a, p<0.001). Similar results were observed in the JE2 strain background, with yoeB mutants demonstrating a lower biofilm bacterial burden with a mean of $5.58\times 10^{5}$ for yoeB1 and a mean of $5.25\times 10^{5}$ for yoeB2 in comparison to $3.0\times 10^{6}$ CFU/mL for the WT (Fig. 2b, p<0.001).

**Figure 3:**
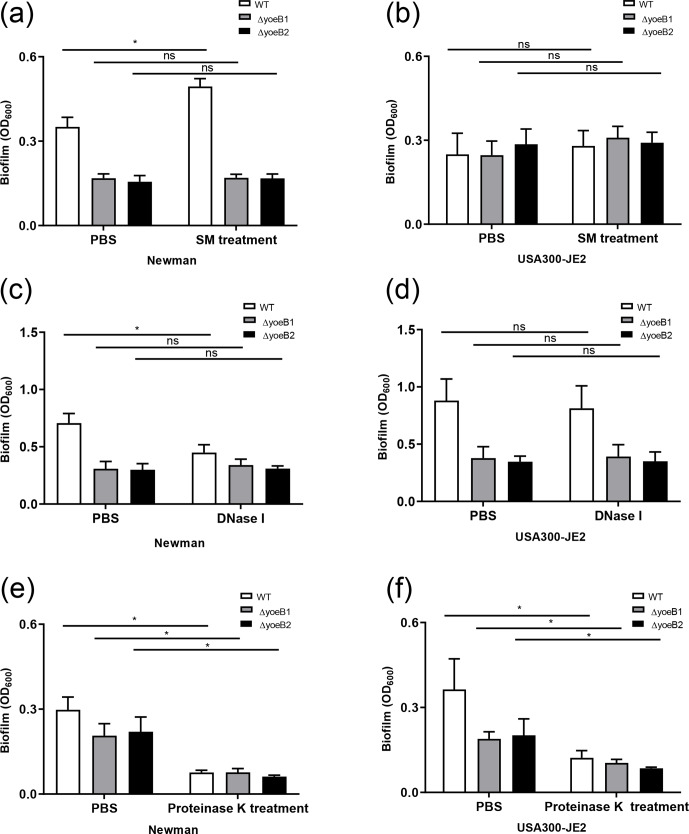
YoeB is associated with extracellular polymeric substance (EPS) biofilm formation. In the **(a)** Newman strain, sodium metaperiodate (SM) treatment of yoeB mutants has no effect on biofilm formation but increases biofilm in WT. No differences were observed in **(b)** USA300-JE2. After DNase I treatment **(c)**, Newman WT demonstrates a reduction in biofilm. However, no changes were observed in yoeB mutants. No differences in biofilm were observed in **(d)** USA300-JE2. Reductions in biofilm formation were observed after proteinase K treatment for **(e)** Newman and **(f)** USA300-JE2 (ns: not significant, * p<0.05).

### 3.3 Biofilm PIA quantitation and changes in biofilm formation after sodium metaperiodate treatment

PIA content in yoeB mutants in the Newman strain background was much lower in comparison to WT (mean 22 325, 100 %), with a mean of 14 001 for yoeB1 (62.7 % of WT) and 4567 for yoeB2 (20.5 % of WT) (Fig. S1a, p<0.001). Similar findings for PIA content were observed in the JE2 strain background in comparison to WT (mean 23 481, 100 %), with a mean of 4255 for yoeB1 (18.1 % of WT) and 839 for yoeB2 (3.6 % of WT) (Fig. S1b, p<0.001). When biofilms were treated with sodium metaperiodate, no reduction in biofilm was observed in yoeB mutants for both Newman (OD${}_{600}$ WT 0.35 yoeB1 0.17, yoeB2 0.17) and JE2 strain backgrounds (OD${}_{600}$ WT 0.28, yoeB1 0.31, yoeB2 0.29) (Fig. 3a and b). However, an increase in biofilm in the Newman WT strain was observed after treatment with sodium metaperiodate (OD${}_{600}$ WT 0.35 before treatment, 0.49 after treatment) (Fig. 3a, p=0.002).

### 3.4 Biofilm eDNA quantitation and changes in biofilm formation after DNase treatment

More eDNA release was observed in the Newman WT strain (100 %) in comparison to yoeB mutants (yoeB1 27 % of WT, yoeB2 25 % of WT) (Fig. S2a, p=0.002). In the JE2 strain background, no differences in eDNA were observed between WT and yoeB mutants (WT 100 %, yoeB1 88 %, yoeB2 103 %) (Fig. S2b). After Newman biofilm were treated with DNase, a reduction was observed in the WT (mean OD${}_{600}$ before treatment 0.71, after treatment 0.45) (Fig. 3c, p<0.001), but no changes were observed in yoeB mutants (mean OD${}_{600}$ before treatment yoeB1 0.31, after treatment 0.34, yoeB2 before treatment 0.30, after treatment 0.31) (Fig. 3c). In JE2, no reduction in biofilm was observed after DNase treatment in the WT (before treatment 0.88, after treatment 0.82) or yoeB mutants (yoeB1 before treatment 0.38, after treatment 0.39, yoeB2 before treatment 0.35, after treatment 0.35) (Fig. 3d).

### 3.5 Biofilm protein quantitation and changes in biofilm formation after proteinase K treatment

In both strain backgrounds, no differences in extracellular protein were observed between WT and yoeB mutants (Newman WT 21.8, yoeB1 24.5, yoeB2 19.3, JE2 WT 21.0, yoeB1 19.1, yoeB2 19.2 µg/mL) (Fig. S3). However, notable decreases in biofilm for both WT and yoeB mutants were observed after treatment with proteinase K in both Newman (before treatment OD${}_{600}$ WT 0.31, yoeB1 0.21, yoeB2 0.22, after treatment WT 0.08, yoeB1 0.08, yoeB2 0.06) and JE2 strain backgrounds (before treatment WT 0.36, yoeB1 0.19, yoeB2 0.20, after treatment WT 0.12, yoeB1 0.10, yoeB2 0.09) (Fig. 3e, p<0.001 and 3f, p<0.001).

### 3.6 Minimum inhibitory concentration (MIC) assay

No differences in MIC were found between WT and yoeB mutants in both strain backgrounds. The MICs for cefazolin and vancomycin in the Newman strain background for both WT and yoeB mutants were 0.38 and 3 µg/mL, respectively. MICs for cefazolin and vancomycin in the JE2 strain background for both WT and yoeB mutants were 1 and 1 µg/mL, respectively.

**Figure 4:**
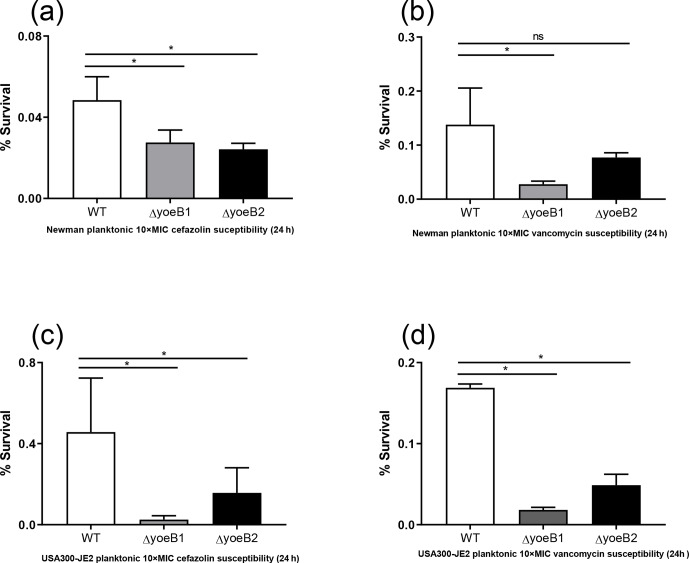
Loss of yoeB1 and yoeB2 increases planktonic antibiotic susceptibility. With the exception of the yoeB2 mutant, reduction of planktonic bacterial survival in yoeB mutants was observed in the Newman strain background for both **(a)** 10X MIC cefazolin and **(b)** 10X MIC vancomycin. Similar results were observed for USA300-JE2, with more notable differences observed in yoeB mutants in comparison to WT for both **(c)** cefazolin and **(d)** vancomycin (ns: not significant, * p<0.05).

### 3.7 Planktonic susceptibility assay

In the Newman strain background, a reduction of planktonic bacterial survival after antibiotic treatment was observed in both yoeB mutants after treatment with 10X MIC cefazolin (OD${}_{600}$ WT 0.05, yoeB1 0.03, yoeB2 0.02) (Fig. 4a, p=0.03, p=0.02). After 10X MIC vancomycin treatment, a decrease in Newman yoeB1 planktonic survival was observed (OD${}_{600}$ WT 0.14, yoeB1 0.03) (Fig. 4b, p<0.001). No differences were observed between WT and yoeB2 (OD${}_{600}$ 0.08) (Fig. 4b). In the JE2 strain background, decreases in planktonic bacterial survival were observed in both yoeB mutants after treatment with cefazolin (OD${}_{600}$ WT 0.46, yoeB1 0.02, yoeB2 0.15) (Fig. 4c, p<0.001, p=0.003) and vancomycin (OD${}_{600}$ WT 0.17, yoeB1 0.02, yoeB2 0.05) (Fig. 4d, p<0.001).

**Figure 5:**
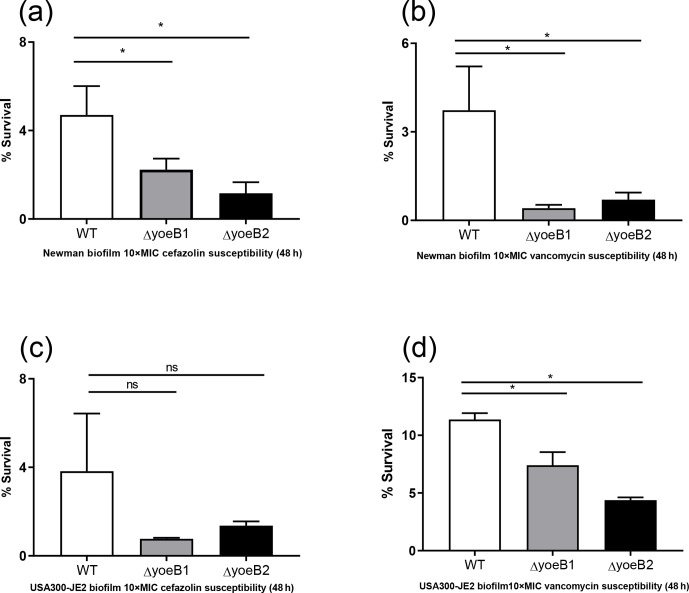
Loss of yoeB1 and yoeB2 increases biofilm antibiotic susceptibility. Deletion of yoeB results in a decrease in biofilm survival in the Newman strain background after treatment with both **(a)** 10X MIC cefazolin and **(b)** 10X MIC vancomycin. No reduction in biofilm survival was observed when JE2 WT and yoeB mutants were treated with **(c)** cefazolin, but differences in biofilm survival were observed between WT and yoeB mutants when biofilms were treated with **(d)** vancomycin (ns: not significant, * p<0.05).

### 3.8 Biofilm susceptibility assay

When biofilms in both strain backgrounds were treated with cefazolin and vancomycin, similar results were observed as in the planktonic assay. Noticeable reductions in yoeB mutants after cefazolin treatment were observed in the Newman strain background (OD${}_{600}$ WT 4.7, yoeB1 2.2, yoeB2 1.2) (Fig. 5a, p<0.001), while no differences were observed in the JE2 strain background (OD${}_{600}$ WT 3.8, yoeB1 0.77, yoeB2 1.4) (Fig. 5c). After treatment with vancomycin, reductions in biofilm for yoeB1 and yoeB2 mutants were observed for both Newman (OD${}_{600}$ WT 3.7, yoeB1 0.41, yoeB2 0.70) (Fig. 5b, p<0.001) and JE2 strain backgrounds (OD${}_{600}$ WT 11.4, yoeB1 7.4, yoeB2 4.4) (Fig. 5d, p=0.001, p<0.001).

### 3.9 In vivo infection in a murine abscess model

**Figure 6:**
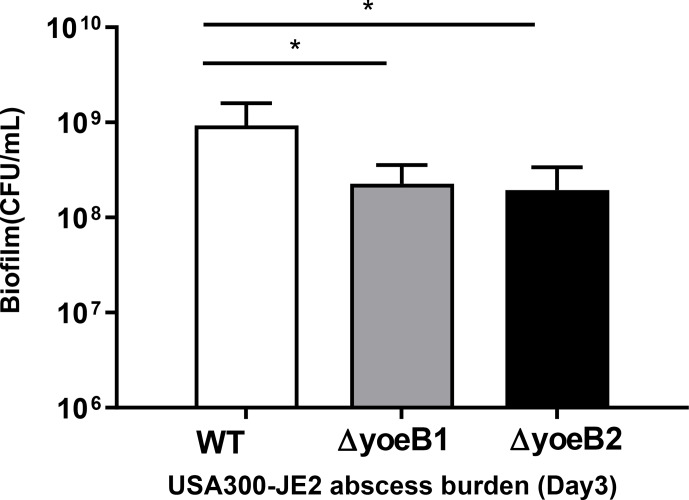
Deletion of yoeB results in reduced bacterial burden in a neutropenic murine abscess model. Bacterial abscess burden in a neutropenic mouse model (n=8 per group) for *S. aureus* USA300-JE2. In comparison to WT, yoeB mutants have a decreased bacterial burden (* p<0.05).

In our neutropenic mouse abscess model, by the third day of infection, WT JE2-infected mice had a mean bacterial burden of $9.26\times 10^{8}$ CFU/mL. Mice infected with yoeB1 and yoeB2 had a decreased abscess burden, with a mean CFU/mL of $2.27\times 10^{8}$ and $1.94\times 10^{8}$ respectively (Fig. 6, p=0.006, p=0.004).

## 4 Discussion

The *S. aureus* type II TA systems have been well studied by our group and others (Schuster and Bertram, 2016; Ma et al., 2019; Chan et al., 2012). However, very little is known about the *S. aureus* YefM–YoeB type II TA system. The objective of this study was to determine the role of yoeB in *S. aureus* planktonic growth, biofilm formation, components of biofilm EPS, antibiotic susceptibility, and virulence.

It is well understood that the toxin–antitoxin complex is separated under environmental stressors (Chan et al., 2012). Under normal growth conditions, the toxin is able to cause growth arrest (Hayes and Van Melderen, 2011; Chan et al., 2012). Bakar et al. (2015) reported that overproduction of yoeB inhibits cell growth and viability (Bakar et al., 2015). We hypothesized that yoeB contributes to bacterial growth. Our results in both strain backgrounds indicate that deletion of both yoeB1 and yoeB2 increases growth of planktonic cells, indicating that the toxin YoeB plays an important role in suppressing planktonic growth.

To investigate the role of yoeB in biofilm formation using clinically relevant materials, we performed a biofilm assay using titanium rods commonly used in orthopaedic surgical procedures. Reductions in biofilm bacterial burden were observed in both yoeB mutants by 72 h of growth. This is in agreement with work by Chan et al. (2018) that demonstrated deletion of yoeB resulted in a reduction in *Streptococcus pneumoniae* biofilm. However, Kato et al. (2017) demonstrated that deletion of yoeB1 and yoeB2 had no effect on biofilm formation. However, this group only looked at biofilms up to 16 h of growth (Kato et al., 2017). Our work focused on a more mature biofilm after 3 d of growth and indicates that YoeB plays an important role in mature biofilm formation.

In order to elucidate the mechanism for decreased biofilm formation in yoeB mutants, we investigated the components of biofilm extracellular polymeric substances (EPS): polysaccharide (PIA), extracellular DNA (eDNA), and protein (Flemming and Wingender, 2010).

Because PIA has been widely studied in relation to biofilm formation (Rohde et al., 2010; Dotto et al., 2017; You et al., 2014), we quantified the biofilm-associated PIA. However, it is unclear whether PIA production is associated with yoeB in *S. aureus*. Similar to previous studies (Rohde et al., 2010; Dotto et al., 2017; You et al., 2014), *S. aureus* WT had a greater PIA concentration in comparison to yoeB mutants in both strain backgrounds. When we compared the corresponding biofilm after treatment with the oxidant sodium metaperiodate, no differences in biofilm were observed in yoeB mutants. Thus, it can only be concluded that in the absence of yoeB, there is a decrease in polysaccharide production, with no correlation in regards to biofilm formation. Interestingly, we did observe an increase in biofilm formation only in the Newman WT strain after treatment with sodium metaperiodate. These results suggest the increase in biofilm may be a response to oxidative stress specific to MSSA strain backgrounds.

Extracellular DNA is another important component of biofilm EPS. Work by other groups has demonstrated that the eDNA component of EPS is essential for biofilm formation (Flemming and Wingender, 2010; Okshevsky and Meyer, 2015) and plays an important role in biofilm architecture in the early stages of infection (Pakkulnan et al., 2019). Previous studies have demonstrated that cellular lysis and the *S. aureus* murein hydrolase regulator contribute to eDNA release (Gao et al., 2013; Rice et al., 2007). eDNA has been reported to serve as a net to hold bacterial cells together, leading to large aggregate formation (DeFrancesco et al., 2017). Other groups have identified that eDNA plays a critical role in biofilm formation but only in the very early stages (Pakkulnan et al., 2019). Interestingly, in the Newman strain, we observed that yoeB mutant biofilms had a much lower eDNA content in comparison to WT, suggesting YoeB may play role in eDNA production, even at the later stages of biofilm formation. Additional work is needed to confirm this phenotype. No differences were observed in the MRSA USA300 JE2 background, indicating possible differences in TA phenotypes between MSSA and MRSA strains that warrant further study. Our results imply *S. aureus* yoeB may be involved in the release of eDNA, which contributes to eDNA-associated biofilm formation. Because of this, we analyzed biofilm formation before and after treatment with DNase. When Newman WT biofilms were treated with DNase, a reduction in biofilm occurred, but no changes in yoeB mutants were observed. Our results demonstrate that YoeB plays a role in biofilm eDNA, but its role in biofilm formation requires further study.

The extracellular-protein-associated biofilm between WT and yoeB mutants was quantified as a final measure of biofilm EPS. No significant differences in extracellular protein concentration were found between WT and yoeB mutants for both Newman and JE2 strain backgrounds. Previous studies have demonstrated that proteinase K treatment leads to a dramatic reduction in *Staphylococcus aureus* biofilms (You et al., 2014; Goormaghtigh et al., 2018). We observed similar findings in our studies for both WT and yoeB mutants, with a reduction in biofilms after treatment with proteinase K in both strain backgrounds. Given our protein and quantitation and biofilm results after proteinase K treatment, it can be concluded that yoeB has no effect on the production of biofilm extracellular protein but does play a role in biofilm formation.

Previous groups have found a correlation between TA genes and changes in MIC (Hemati et al., 2014; Wen et al., 2014). In order to determine if yoeB1 and yoeB2 are involved in antibiotic tolerance, we determined the cefazolin and vancomycin MIC of WT and yoeB mutants in both strain backgrounds. Similar to our previous work with *S. aureus* type II TA systems (Ma et al., 2019), no differences in MIC were observed between WT and yoeB mutants during stationary phase growth.

Since TA systems have been demonstrated to play a role in response to planktonic antibiotic stress (Ma et al., 2019; Goormaghtigh et al., 2018; Salzberg and Helmann, 2007; Costa et al., 2009), the role of yoeB in antibiotic susceptibility was explored. Loss of yoeB1 and yoeB2 resulted in increased susceptibility to cefazolin in both strain backgrounds. When comparing strain backgrounds for vancomycin treatment, susceptibility was only observed for yoeB1 in Newman, while vancomycin susceptibility was observed in both yoeB mutants for JE2, indicating possible differences between MSSA and MRSA strain backgrounds.

It is still unclear whether TA systems affect biofilm formation under conditions of antibiotic stress. Previous work indicates that deletion of type II TA systems leads to decreased antibiotic resistance in *Escherichia coli*; however, this work did not determine a link between the induction of TA systems and antibiotic resistance (Goormaghtigh et al., 2018). Salzberg and Helmann (2007) demonstrated that yoeB is a cell-wall-associated gene that is able to protect *Bacillus subtilis* from cell-wall-targeted antibiotic stress. Our previous work in another *S. aureus* type II TA system demonstrated that the toxin component of the system is susceptible to antibiotics (Ma et al., 2019). This is in agreement with our current results, where yoeB toxin mutants are more susceptible to cell-wall-targeting antibiotics under biofilm growth conditions.

Finally, we investigated the role of yoeB in the context of a live host. *S. aureus* infections are not chronic, unless the infection has developed over an extended period of time such as in surgical cases. In these types of infection, biofilms play a critical role (Urish et al., 2018; Ma et al., 2018). Bacterial growth, antibiotic susceptibility, and biofilm formation are all crucial for the establishment of infection and virulence (Costerton, 1999; Donlan, 2001). We used a neutropenic mouse abscess model and quantified bacterial burden as a measure of virulence, as has been demonstrated in other studies (Kobayashi et al., 2015; Tram et al., 2018). Loss of both yoeB1 and yoeB2 resulted in decreased bacterial burden in our mouse abscess model. In addition, bacteria in the infected tissue samples were predominantly in the biofilm phenotype. These findings were similar to our in vitro biofilm data but are in contrast to our previous findings in another *S. aureus* type II TA system (Ma et al., 2019), suggesting a different role of the YefM–YoeB TA system in virulence. In addition, our animal data conflict with our planktonic growth results, where we observed increased growth in yoeB mutants over time. Additional work is needed to identify the mechanism of the phenotypic differences between planktonic and biofilm forms of growth.

There were some limitations to these studies. With the exception of the neutropenic mouse model, all other experiments were completed in vitro. Thus, it is difficult to compare these results to a clinical scenario. The interaction of microbial growth, nutrient utilization, nutrient diffusion, biofilm formation, antibiotic killing, and development of resistance is complex. In addition, rifampicin is widely used in combination with other antibiotics for staphylococci biofilm infections (Zimmerli and Sendi, 2019) and is the focus of future studies used in combination with vancomycin and cefazolin. The MIC values used to test biofilm susceptibility to antibiotics were determined in bacteria grown in the planktonic state only. A more effective measure of biofilm susceptibility would be to test antibiotic concentrations at the minimum biofilm inhibitory concentration. However, our data did demonstrate differences between WT and yoeB mutants under the currently tested conditions after 48 h of growth. Our current results show contrasting differences between our animal results, biofilm formation, and the planktonic cell growth; therefore more work is warranted to identify the TA mechanism of these phenotypic differences.

Our current data demonstrate the importance of the YefM–YoeB toxin–antitoxin system in planktonic growth, biofilm formation, components of biofilm EPS, planktonic antibiotic tolerance, and biofilm antibiotic tolerance. Our data also highlight the importance of toxin–antitoxin systems in infection, but the role of YefM–YoeB in virulence and infection progression requires further study.

## Supplement

10.5194/jbji-6-241-2021-supplement The supplement related to this article is available online at: https://doi.org/10.5194/jbji-6-241-2021-supplement.

## Data Availability

For access to raw data, contact the corresponding author (Kenneth L. Urish: urishk2@upmc.edu).

## Code availability

Software code used for statistical analysis is available upon reasonable request from the corresponding author.

## Appendix

The supplement related to this article is available online at: https://doi.org/10.5194/jbji-6-241-2021-supplement.
