# Supplementary material for: The Staphylococcus aureus toxin–antitoxin system YefM–YoeB is associated with antibiotic tolerance and extracellular dependent biofilm formation
Source: J Bone Jt Infect. 2021 Jul 2;6(7):241–53. doi: 10.5194/jbji-6-241-2021 (PMC8273624; doi:10.5194/jbji-6-241-2021)
Supplement: The supplement related to this article is available online at: https://doi.org/10.5194/jbji-6-241-2021-supplement. [file jbji-6-241-supplement.pdf]

Supplement of J. Bone Joint Infect., 6, 241–253, 2021
https://doi.org/10.5194/jbji-6-241-2021-supplement
© Author(s) 2021. CC BY 4.0 License.

Supplement of

The *Staphylococcus aureus* toxin–antitoxin system YefM–YoeB is associated with antibiotic tolerance and extracellular dependent biofilm formation

Xinyu Qi et al.

Correspondence to: Kenneth L. Urish (urishk2@upmc.edu)

The copyright of individual parts of the supplement might differ from the article licence.

Figure S1. Loss of *yoeB* results in reduced PIA. Chemidoc image analysis of biofilm PIA content for (a) Newman and (b) USA300-JE2 strain backgrounds. (*$p<0.05$)

Figure S2. Loss of *yoeB* results in Newman reduced biofilm associated eDNA. Biofilm percent eDNA relative to WT for (a) Newman and (b) USA300-JE2 strain backgrounds. (ns = not significant, *$p<0.05$).

Figure S3. Biofilm protein content for *S. aureus* strains quantified by Bicinchoninic Acid (BCA) (a) Newman and (b) USA300-JE2. (ns = not significant)

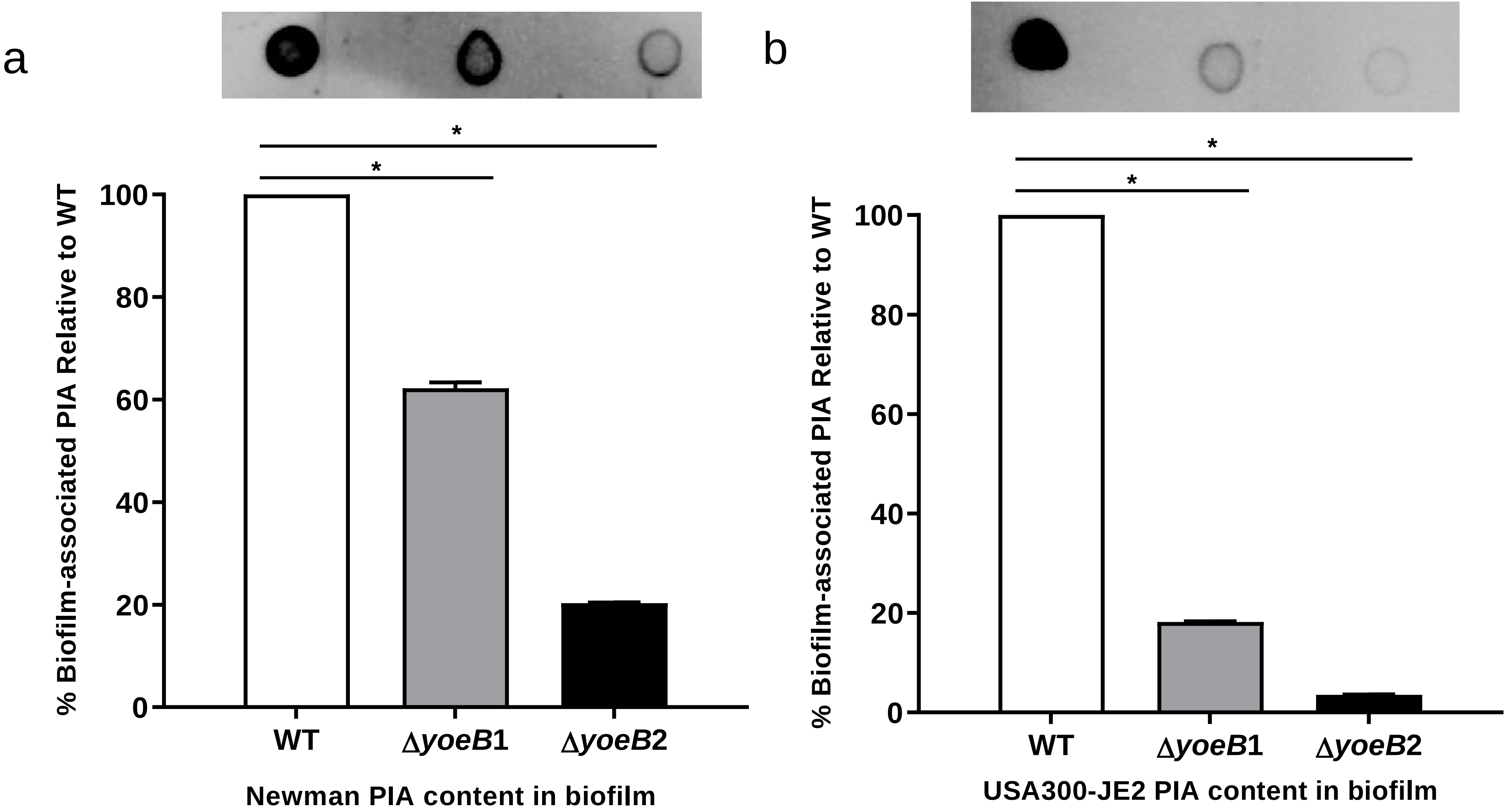

Figure S1. Loss of *yoeB* results in reduced PIA. Chemidoc image analysis of biofilm PIA content for (a) Newman and (b) USA300-JE2 strain backgrounds. (*p<0.05)

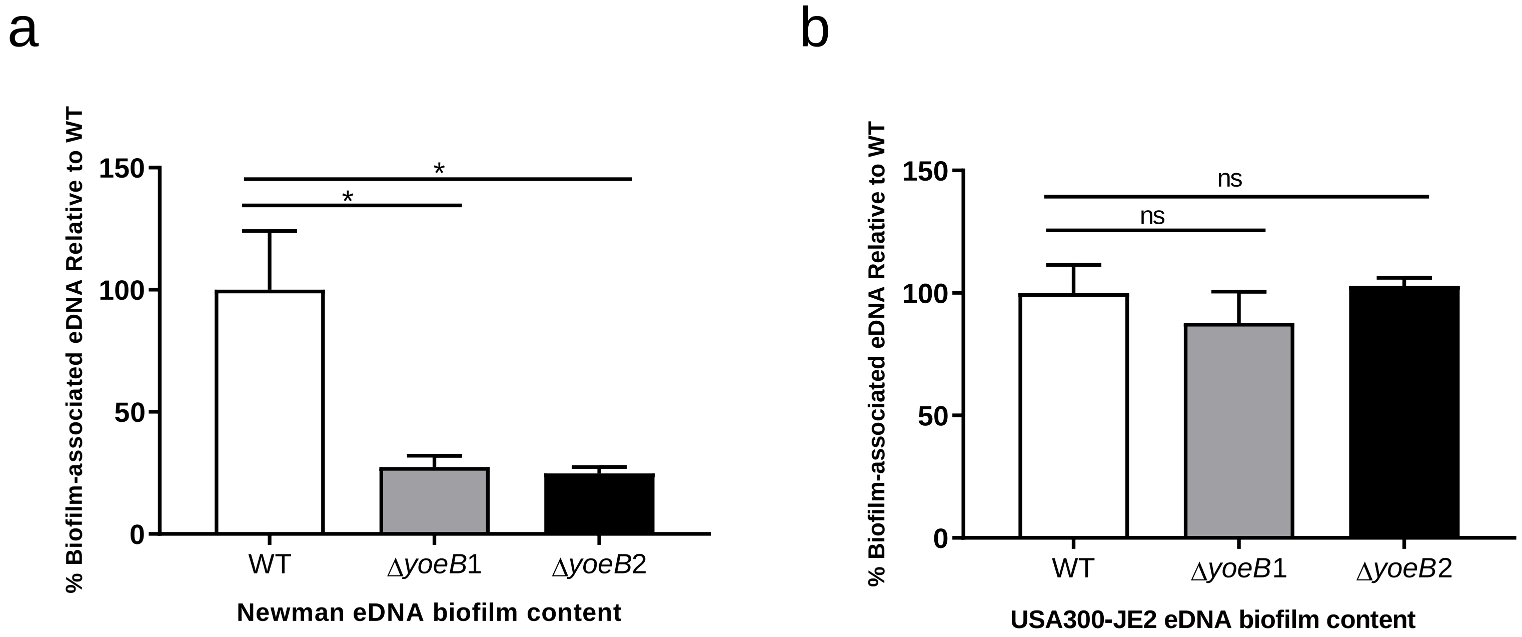

Figure S2. Loss of *yoeB* results in Newman reduced biofilm associated eDNA. Biofilm percent eDNA relative to WT for (a) Newman and (b) USA300-JE2 strain backgrounds. (ns = not significant, *p<0.05)

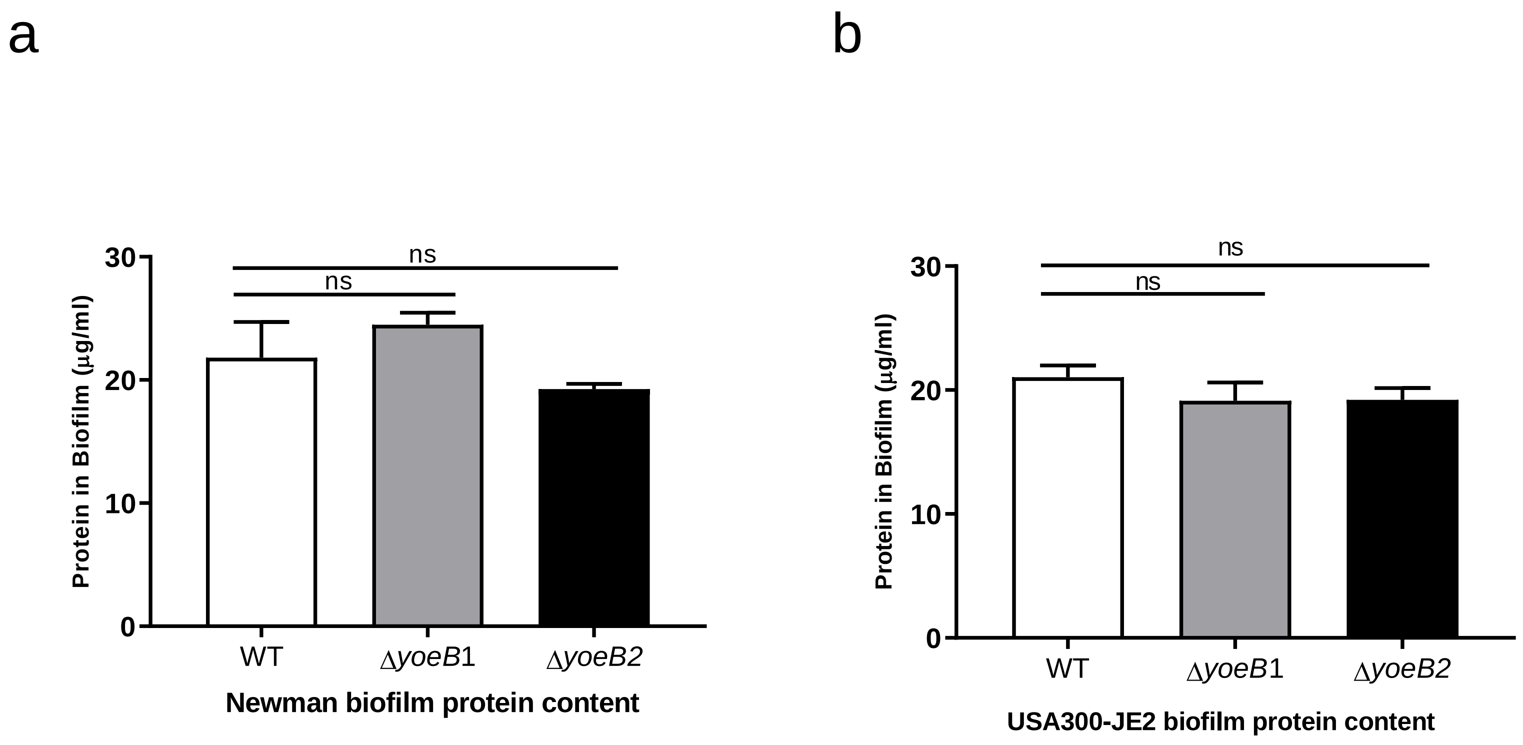

Figure S3. Biofilm protein content for *S. aureus* strains quantified by Bicinchoninic Acid (BCA) (a) Newman and (b) USA300-JE2. (ns = not significant)
